# Magnesium Enhances Osteogenesis of BMSCs by Tuning Osteoimmunomodulation

**DOI:** 10.1155/2019/7908205

**Published:** 2019-11-14

**Authors:** Xufang Zhang, Qingpiao Chen, Xueli Mao

**Affiliations:** ^1^Department of Operative Dentistry and Endodontics, Guanghua School of Stomatology, Guangdong Province Key Laboratory of Stomatology, Sun Yat-sen University, Guangzhou 510055, China; ^2^Department of General Dentistry and Oral Health, Guanghua School of Stomatology, Guangdong Province Key Laboratory of Stomatology, Sun Yat-sen University, Guangzhou 510055, China

## Abstract

In the process of bone tissue engineering, the osteoimmunomodulatory property of biomaterials is very important for osteogenic differentiation of stem cells, which determines the outcome of bone regeneration. Magnesium (Mg) is a biodegradable, biocompatible metal that has osteoconductive properties and has been regarded as a promising bone biomaterial. However, the high degradation rate of Mg leads to excessive inflammation, thereby restricting its application in bone tissue engineering. Importantly, different coatings or magnesium alloys have been utilized to lower the rate of degradation. In fact, a prior study proved that *β*-TCP coating of Mg scaffolds can modulate the osteoimmunomodulatory properties of Mg-based biomaterials and create a favorable immune microenvironment for osteogenesis. However, the osteoimmunomodulatory properties of Mg ions themselves have not been explored yet. In this study, the osteoimmunomodulatory properties of Mg ions with involvement of macrophages and bone marrow stem cells (BMSCs) were systematically investigated. Microscale Mg ions (100 mg/L) were found to possess osteoimmunomodulatory properties that favor bone formation. Specifically, microscale Mg ions induced M2 phenotype changes of macrophages and the release of anti-inflammatory cytokines by inhibiting the TLR-NF-*κ*B signaling pathway. Microscale Mg ions also stimulated the expression of osteoinductive molecules in macrophages while Mg ions/macrophage-conditioned medium promoted osteogenesis of BMSCs through the BMP/SMAD signaling pathway. These findings indicate that manipulating Mg ion concentration can endow the Mg biomaterial with favorable osteoimmunomodulatory properties, thereby providing fundamental evidence for improving and modifying the effect of Mg-based bone biomaterials.

## 1. Introduction

Foreign materials for repairing bone defects have a great influence on osteogenesis and osteoclasts, forming the basis for the study of osteoimmunology. Osteoimmunology aims to understand the interaction and related mechanism between the skeletal system and immune system [1]. When an implant is placed into a host, immune response around the implant is triggered. Following the start of immune response, phenotype switching of macrophages and adhesion of interleukin- (IL-) 10, IL-1ra, and other inflammatory factors occur, which also have an influence on cells associated with osteogenesis and osteoclasts [1, 2]. As there is a strong relationship between the immune system and the skeletal system, an ideal bone biomaterial in the host should be able to accelerate osteogenesis in the bone defect area through local immune response. Immunomodulatory properties of bone substitute materials are suggested to be of great importance for the success of bone tissue engineering [1, 2].

Magnesium (Mg) is an essential inorganic component in bone tissue and plays an important role in skeletal development. Mg has mechanical properties similar to those of bone tissue and displays antibacterial activity, excellent biocompatibility, and biodegradability [3, 4]. Studies have shown that Mg ion supplementation improved the adhesion of osteoblasts to biomaterials, mediated by integrin [5]. In addition, Mg ions act as the nuclei for hydroxyapatite formation to promote bone matrix mineralization [6, 7]. Mg-incorporation of mesoporous TiO_2_ coatings showed better surface, osteoconductive ability, and elevated expression of osteogenic genes [8]. However, there is still a great challenge that must be addressed before Mg can be utilized clinically. The active chemical character of Mg will not only produce a great amount of air, which reduces the contact between bone and material, but also result in an inflammatory reaction due to rapid degradation. Notably, different coatings or magnesium alloys have been utilized to lower the rate of degradation. A prior study showed that *β*-TCP coating of Mg scaffolds can modulate the scaffold's osteoimmunomodulatory properties and shift the immune microenvironment toward one that favors osteogenesis over osteoclastogenesis [9]. However, the osteoimmunomodulatory properties of Mg ion itself have not yet been explored.

Macrophages play an important role in human immune defense and osteoimmunology [10, 11]. There are two typical phenotypes of macrophages. The classically activated M1 phenotype mainly participates in T helper cell 1- (Th1-) type inflammation, which is involved in defense against foreign harmful substances, but can sometimes cause excessive inflammatory response in host. Additionally, the alternatively activated M2 phenotype is involved in Th2-type inflammation which reduces inflammation response and improves impairment [12, 13]. These two phenotypes can switch to each other in response to biomaterials or microbes. Furthermore, following phenotype switching of macrophages, osteoinductive molecules such as bone morphogenetic protein 2 (BMP-2) and transforming growth factor-*β* (TGF-*β*) can be secreted to promote osteogenesis [14, 15]. Given their important roles in bone remodeling, the response of macrophages was applied to evaluate the osteoimmunomodulatory properties of biomaterials [16].

In the present study, the osteoimmunomodulatory properties of microscale Mg ions were extensively investigated by using a biomimicking condition comprising Mg ions, bone marrow stem cells (BMSCs), and macrophages. First, the phenotype changes of macrophages in response to Mg ions and inflammatory/anti-inflammatory cytokines were evaluated to assess the immune environment. Thereafter, the important inflammatory signaling pathway factor, nuclear factor kappa B (NF-*κ*B), was studied to explore the molecular mechanism of Mg ions in macrophages. The osteogenic differentiation of BMSCs mediated by the Mg ions was then investigated under the influence of macrophages, to prove whether the regulated immune environment by Mg ion could promote osteogenesis. The aim of this study was to determine whether microscale Mg ions possess osteoimmunomodulatory properties and whether this regulated immune environment could positively influence osteogenesis, ultimately providing the fundamental evidence of utilizing Mg-based biomaterial as bone scaffold.

## 2. Materials and Methods

### 2.1. Cell Culture

The murine-derived macrophage cell line, RAW 264.7 (RAW), was cultured in Dulbecco's modified Eagle Medium (DMEM, Gibco, USA) supplemented with 10% heat inactivated fetal bovine serum (FBS, Gibco) and 1% (v/v) penicillin/streptomycin (Sigma, USA) at 37°C in a humidified CO_2_ incubator. Growing cells were expanded for two passages before use in this study. Mice BMSCs were isolated and cultured following protocols from previous studies [9]. Bone marrow was briefly isolated from SD mice (5–6 weeks old). Under aseptic conditions, bilateral femurs and tibias of rats were isolated and removed. Bone marrow was rinsed with DMEM solution and centrifuged at 1000 rpm for 5 min. The supernatant was discarded, and the precipitate was resuspended with culture medium containing DMEM, 15% FBS, and 1% (v/v) penicillin/streptomycin. Cells were then seeded in tissue culture flasks and incubated at 37°C in a humidified CO_2_ incubator. The culture medium was first changed within 24 h and then every 3 days. The attached cells were expanded and early passages (p3) were used in the following study.

### 2.2. Effect of Mg Ions on RAW264.7 Cells

#### 2.2.1. Proliferation of RAW Cells Stimulated with Mg Ions

Cell culture medium consisted of DMEM without Mg ions (HyClone, USA), 10% FBS, and 1% (v/v) penicillin/streptomycin. Based on the molecular weights of Mg, S, and O, MgSO_4_ was added to the culture medium to a final concentration of 0, 5, 10, 25, 50, 100, 250, and 500 mg/L Mg ions. Additionally, the effects of Mg ions on RAW proliferation were investigated using Cell Counting Kit-8 Assay (CCK-8, Dojindo, Japan). RAW cells were seeded in 96-well microplates at a density of 2 × 10^3^ cells/well and were allowed to adhere and spread for 24 h. RAW cells were then treated with various concentrations of Mg ions (0, 5, 10, 25, 50, 100, 250, and 500 mg/L) for 1, 3, and 7 days. Thereafter, cells were incubated with the CCK-8 solution for 2 h. The absorbance was measured at 450 nm by using a UV spectrophotometer.

#### 2.2.2. Phenotype Switches and Expression of Inflammatory Genes in RAW Cells

Gene expression of macrophage surface markers (CCR7 and CD206) and inflammatory-related cytokines (IL-1ra, IL-10, IL-1*β*, IL-6, IL-18, and TNF-*α*) were detected by RT-PCR to observe the phenotype changes and pro/anti-inflammation ability of RAW cells. These cells were also seeded on 6-well plates at a density of 1 × 10^6^ cells/well. LPS (1 *μ*g/mL) was added to the media when it reached 80% confluence to activate RAW cells for 2 h. Cells were then stimulated with different concentrations of Mg ions (0, 10, 100, and 500 mg/L) for 6 h. Thereafter, total RNA was extracted by using TRIzol reagent (Invitrogen, USA), and the RNA concentrations were quantified with a Nanodrop protein/nucleic acid spectrophotometer (Thermo-Fisher, USA). Notably, first strand cDNA was synthesized using the RNA reverse transcription kit (Takara, Japan), and qRT-PCR was performed using a SYBR Green I Master kit (Takara) in LightCycle 96 RT-PCR (Roche, Switzerland). Primers for the target genes are listed in Table 1.

#### 2.2.3. Activation of Toll-Like Receptor (TLR) and NF-*κ*B Signaling Pathways in RAW Cells

The TLR and NF-*κ*B pathways were analyzed to explore the molecular mechanisms that underlie the macrophage gene changes. RAW cells were seeded on 6-well plates at a density of 1 × 10^6^ cells per well and grew to 80% confluence. RAW cells were first activated by LPS (1 *μ*g/mL) for 2 h and then stimulated with different concentrations of Mg ions for 6 h. Total RNA was collected for gene detection of myeloid differentiation factor 88 (MyD88) and TIR domain-containing adapter molecule 1 (Ticam1) and Ticam2 by RT-PCR, using the same method described in Section 2.2.2.

Whole cell lysates were also collected after 6 h of stimulation by Mg ions, and protein expression of NF-*κ*B p65 and inhibitor protein kappa B (I-*κ*B) were determined by western blot. In addition, total protein from RAW cells was extracted using the total protein extraction reagent kit (Beyotime Institute, Shanghai, China) and protein concentration was measured using the BCA assay. Equal amounts of protein (20 *μ*g) were prepared and separated using 10% sodium dodecyl sulphate polyacrylamide gel electrophoresis (SDS-PAGE) and then transferred onto PVDF membranes. Membranes were blocked in TBST containing 50 g/L skim milk powder for 2 h and incubated with primary antibodies overnight at 4°C. The primary antibodies included rabbit against mice anti-p-I-*κ*B polyclonal antibody (Bioss Corporation, Beijing, China), anti-NF-*κ*B p65 monoclonal antibody (Santa Cruz, USA), and anti-GAPDH (Abcam, UK). Membranes were then washed 3 times and probed with the secondary antibody, anti-rabbit IgG (Bioteke Corporation, Beijing, China). The results were detected with the ECL detection kit, and the relative intensity of protein bands was quantified using the Image J software. Levels of I-*κ*B and NF-*κ*B expression were calculated relative to GADPH.

#### 2.2.4. Expression of Osteogenesis-Related Cytokines in RAW Cells

RAW cells were seeded on 6-well plates, activated by LPS for 2 h, and stimulated with different concentrations of Mg ions for 6 h as described in Section 2.2.3. Samples were collected and subjected to RT-PCR for the detection of BMP-2, BMP-6, TGF-*β*1, TGF-*β*3, and vascular endothelial growth factor (VEGF), using the method described in Section 2.2.2.

### 2.3. Effects of Mg Ions/RAW Cells-Conditioned Media on the Osteogenic Differentiation of BMSCs

#### 2.3.1. ALP Activity Test

RAW cells were stimulated with different concentrations of Mg ions (0, 10, 100, and 500 mg/L) for 6 h. Culture media were then collected and marked as Mg ions/RAW264.7 cells-conditioned media. Alkaline phosphatase (ALP) activity of BMSCs in the condition medium was measured using the Alkaline Phosphatase Assay Kit (BioAssaySystems, USA). BMSCs were seeded on 24-well plates at a density of 5 × 10^4^ cells per well with the complete culture medium. After 80% confluence, cells were stimulated with Mg ions/RAW264.7 cells-conditioned media for 7 days. Cells were then lysed in 1% Triton X-100. The supernatant of the medium was then harvested for ALP assay, and optical density (OD) was detected at 405 nm with a spectrophotometer. ALP activity is presented as OD values divided by the reaction time and total protein amount.

#### 2.3.2. Osteogenic Gene Expression of BMSCs

BMSCs were seeded on 24-well plates at a density of 5 × 10^4^ cells per well with the complete culture medium. BMSCs were also stimulated with Mg ions/RAW264.7 cells-conditioned media for 1 day and 3 days. Samples were collected and subjected to RT-PCR for the detection of the osteogenic genes, Runx-2, ALP, OPN, and OCN, with the method described in Section 2.2.2.

#### 2.3.3. Activation of BMP/SMAD Signaling Pathway in BMSCs

BMSCs were stimulated with Mg ions/RAW264.7 cells-conditioned media for 1 and 3 days, and total RNA was extracted to study the activation of BMP/SMAD pathway by RT-PCR as described in Section 2.2.2. Pathway-related genes included mothers against decapentaplegic homolog 1/4/5 (SMAD1/4/5) and bone morphogenetic protein receptor type IA (BMPR1A). Protein levels of SMAD4 and BMPR1A were further confirmed by western blot on days 3 and 7. The detailed methods are described in Section 2.2.3.

#### 2.3.4. Statistical Analysis

All data were presented as mean ± standard deviation (SD). Statistical analyses were performed with SPSS 22.0. One-way analysis of variance (ANOVA) was used to analyze the statistical difference when more than 2 groups were compared. Student's *t*-test was used to compare experimental groups and control group. A *P* value <0.05 was considered to be statistically significant.

## 3. Results

### 3.1. Effect of Mg Ions on Cell Proliferation of Macrophages

**Figure 1:**
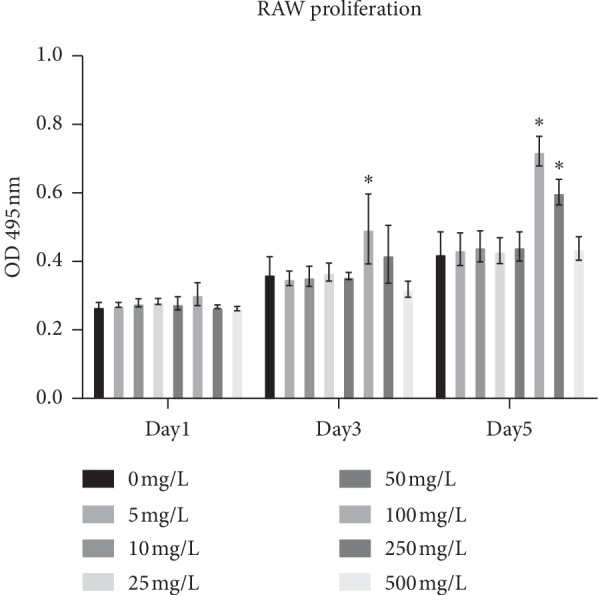
Effect of Mg ions on the proliferation of RAW264.7 cells. ^*∗*^*P* < 0.05 versus the control without Mg ions.

To identify the cytotoxic effects of Mg ions, macrophages were treated with different concentrations of Mg ions (5, 10, 25, 50, 250, and 500 mg/L) for 1, 3, and 5 days (Figure 1). The CCK-8 assay showed that 100 mg/L (<100 mg/L) Mg ions had no obvious influence on the proliferation of RAW264.7 cells compared to control (*P* > 0.05). However, Mg ions at a concentration of 100 mg/L significantly increased the proliferation of RAW264.7 on days 3 and 5 (*P* < 0.05). On day 5, cell proliferation also significantly increased at a concentration of 250 mg/L (*P* < 0.05).

### 3.2. Surface Marker Changes and Inflammatory Gene Expression of RAW Cells in response to Mg Ions

**Figure 2:**
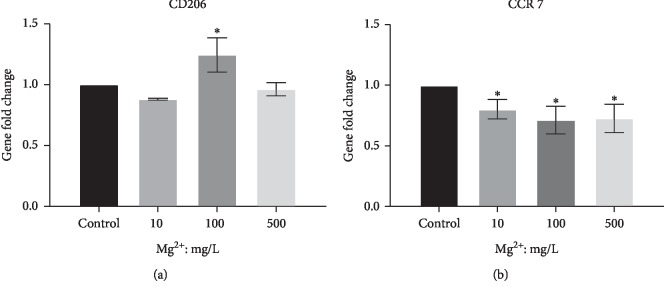
Effect of Mg ions on RAW264.7 phenotype transformation. (a) Gene expression of the M2 phenotype marker, CD206. (b) Gene expression of the M1 phenotype marker, CCR7. ^*∗*^*P* < 0.05, compared to the control group without Mg ions.

Stimulation of RAW cells with Mg ions (100 mg/L) revealed the increased gene expression of the M2 surface marker, CD206, relative to the control group (*P* < 0.05, Figure 2(a)), thereby indicating a shift toward the M2 phenotype in response to Mg ions. In contrast, stimulation with Mg ions resulted in reduced gene expression of the M1 phenotype marker, CCR7, compared to the control group (*P* < 0.05, Figure 2(b)).

**Figure 3:**
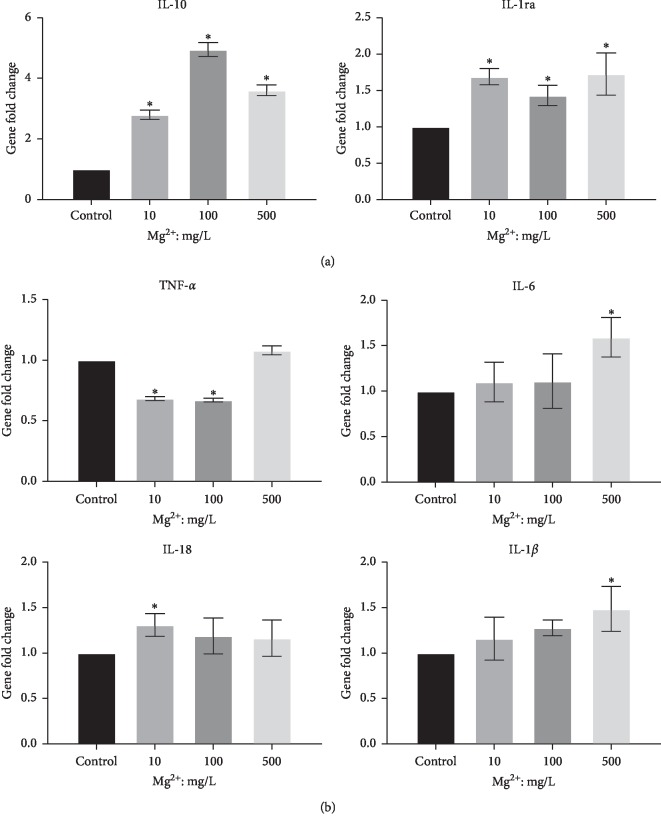
Effect of Mg ions on the gene expression of anti-inflammatory and inflammatory cytokines in RAW264.7 cells. (a) Gene expression of the anti-inflammatory cytokines, IL-10 and IL-1a. (b) Gene expression of the anti-inflammatory cytokines, TNF-*α*, IL-6, IL-18, and IL-1*β*. ^*∗*^*P* < 0.05, compared to the control group without Mg ions.

The gene expression of anti-inflammatory and inflammatory cytokines was detected in RAW264.7 cells after exposure to Mg ions for 6 h. The expression level of anti-inflammatory genes (IL-10 and IL-1ra) was upregulated at all concentrations of Mg ions compared to the control (*P* < 0.05, Figure 3(a)). In contrast, the expression of the inflammatory cytokine, TNF-*α*, was significantly downregulated at concentrations of 10 mg/L and 100 mg/L of Mg ions (*P* < 0.05, Figure 3(b)). Furthermore, the expression of other inflammatory cytokines increased slightly. For instance, the expression of IL-6 and IL-1*β* increased at 500 mg/L Mg ions and that of IL-18 increased at 10 mg/L Mg ions (*P* < 0.05, Figure 3(b)). However, the fold changes of inflammatory cytokines (IL-6, IL-1*β*, and IL-18) were obviously less than that of anti-inflammatory cytokines (IL-10 and IL-1ra).

### 3.3. Effect of Mg Ions on TLRs and NF-*κ*B Signaling Pathway in RAW 264.7 Cells

**Figure 4:**
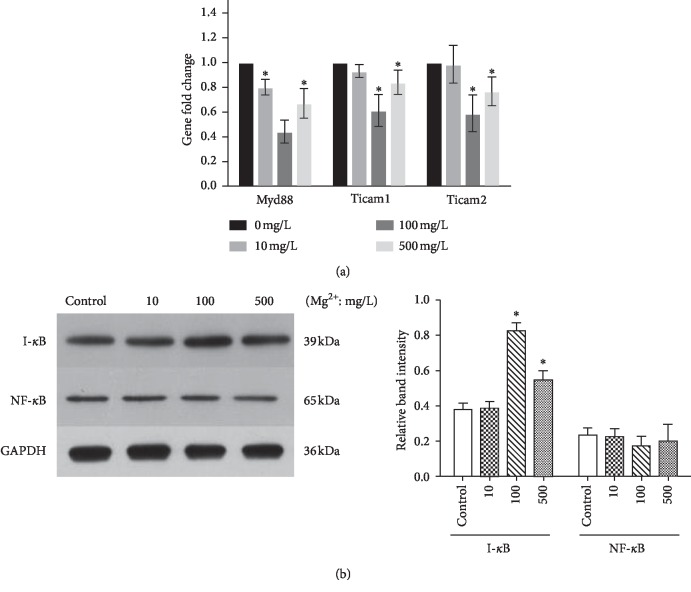
Effect of Mg ions on the TLR and NF-*κ*B signaling pathways of RAW 264.7 cells. (a) Gene expression of the TLRs pathway markers, Myd88, Ticam1, and Ticam2 in RAW264.7 cells. (b) Protein expression of NF-*κ*B p65 and I-*κ*B in RAW 264.7 cells. ^*∗*^*P* < 0.05, compared to the control group without Mg ions.

To explore the molecular mechanisms of the inflammation-related gene alterations, the TLRs and NF-*κ*B signaling pathways were examined in RAW cells. Compared to the control group, gene expression of Myd88, Ticam1, and Ticam2 were downregulated in 100 and 500 mg/L Mg ion groups with significant differences (*P* < 0.05, Figure 4(a)). Western blot also showed that the protein expression of NF-*κ*B p65 had no significant difference between Mg ion and control groups. In contrast, the downstream molecular I*κ*B-*α* was upregulated at 100 and 500 mg/L Mg ions compared to the control (*P* < 0.05, Figure 4(b)), indicating the inhibition of the TLR-NF-*κ*B signaling pathway.

### 3.4. Effect of Mg Ions on the Expression of Osteogenesis-Related Cytokines in RAW264.7 Cells

**Figure 5:**
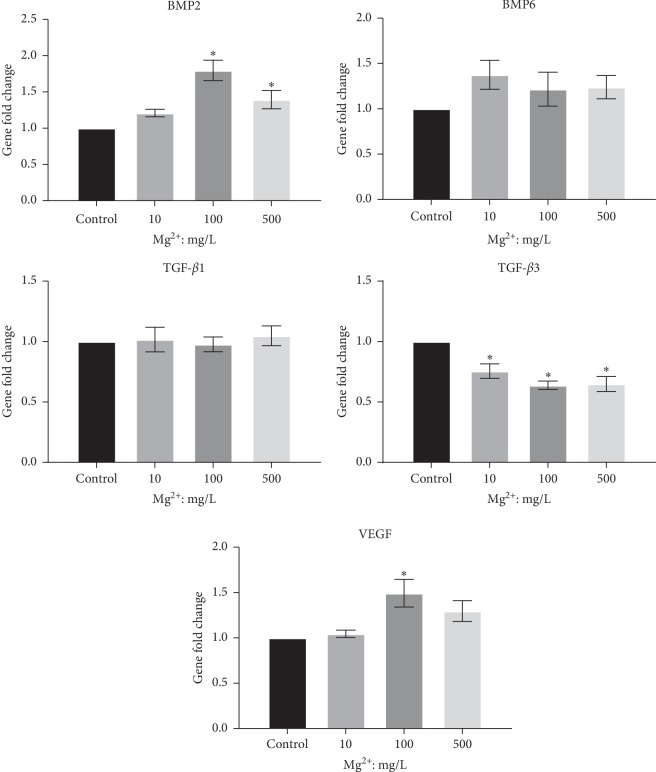
Effect of Mg ions on the gene expression of osteogenesis-related cytokines (BMP2, BMP6, TGF-*β*1, TGF-*β*3, and VEGF) in RAW264.7 cells. ^*∗*^*P* < 0.05, compared to the control group without Mg ions.

BMPs, the TGF-*β* family, and VEGF are all important osteogenesis-related factors. RT-PCR demonstrated that gene levels of BMP-2 and VEGF were significantly higher in 100 mg/L Mg ion groups than that of control (*P* < 0.05, Figure 5). In contrast, TGF-*β*3 gene expression level was slightly downregulated in Mg ion groups compared to the control group (*P* < 0.05). Gene expression of TGF-*β*1 and BMP-6 showed no obvious differences in each group.

### 3.5. Effects of Mg Ions/RAW264.7 Cells-Conditioned Media on the Osteogenic Differentiation of BMSCs

**Figure 6:**
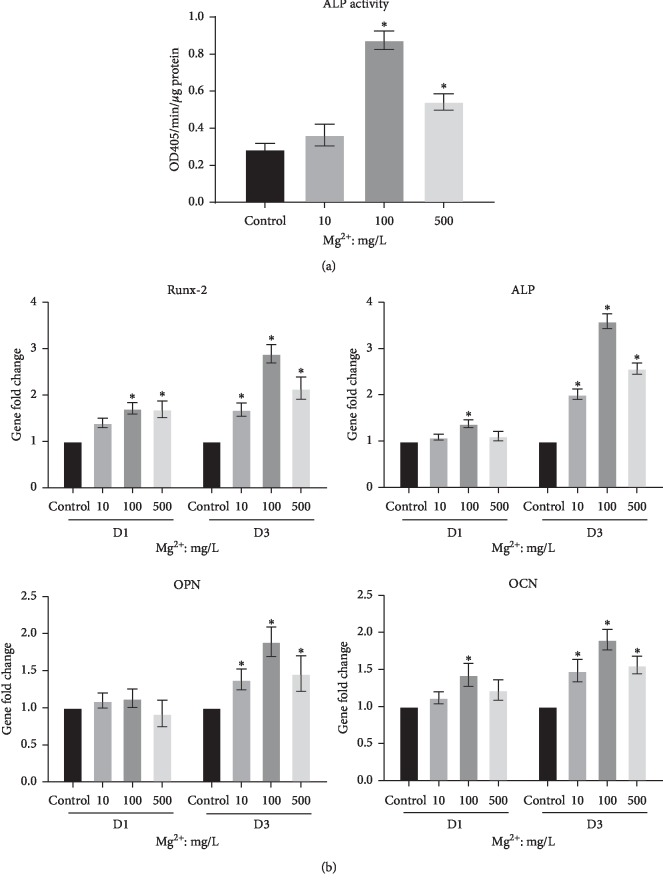
Effects of Mg ions/RAW264.7 cells-conditioned media on the osteogenic differentiation of BMSCs. (a) ALP activity of BMSCs in Mg ions/RAW264.7 cells-conditioned media. (b) Osteogenic gene expression of BMSCs in Mg ions/RAW264.7 cells-conditioned media. ^*∗*^*P* < 0.05, compared to the control group.

To clarify whether Mg ions influence osteogenesis of BMSCs through regulating macrophages, Mg ions/RAW264.7 cells-conditioned media were utilized for osteogenic differentiation of BMSCs. The results showed that when BMSCs were stimulated with conditioned media containing 100 and 500 mg/L Mg ions, ALP activity was significantly enhanced compared to control (*P* < 0.05, Figure 6(a)). Furthermore, osteogenic gene expression of BMSCs in conditioned media was explored by RT-PCR. On day 3, BMSCs stimulated with Mg ions/RAW264.7 cells-conditioned medium of all concentrations of Mg ions had a significantly upregulated expression of the osteogenic genes (Runx-2, ALP, OPN, and OCN) compared to the control group (*P* < 0.05, Figure 6(b)). In addition, on day 1, conditioned medium with 100 mg/L Mg ions significantly increased gene expression of Runx-2, ALP, and OCN in BMSCs (*P* < 0.05, Figure 6(b)).

### 3.6. Activation of the BMP/SMAD Signaling Pathway in BMSCs Stimulated with the Mg Ion/RAW264.7 Cells-Conditioned Media

**Figure 7:**
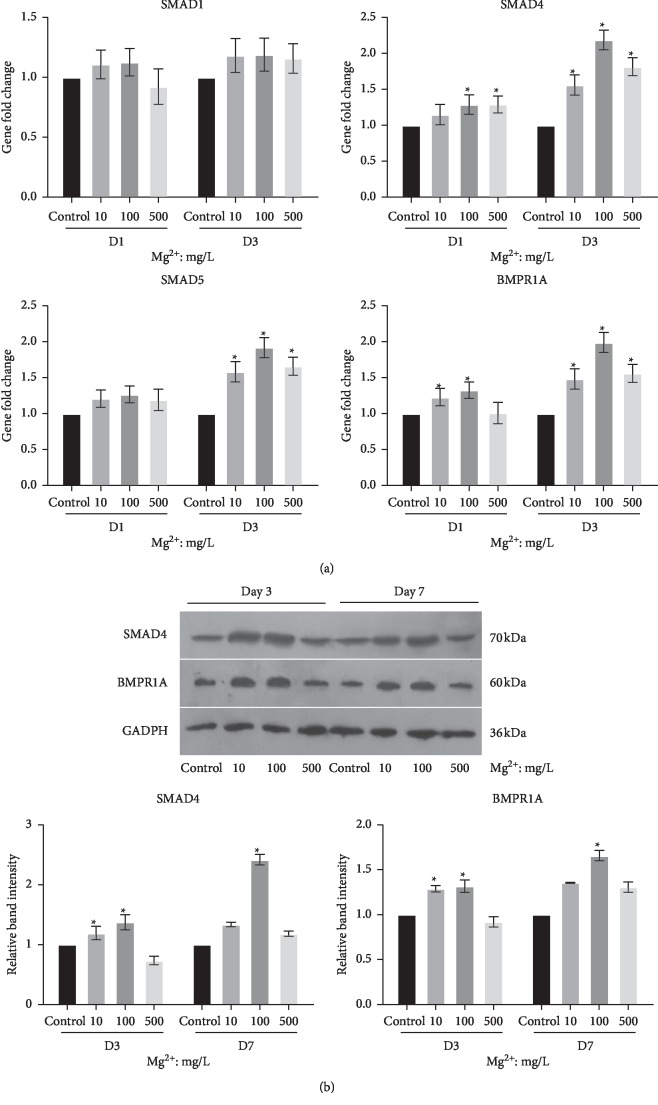
Activation of the BMP/SMAD signaling pathway in BMSCs stimulated with the Mg ions/RAW264.7 cells-conditioned media. (a) Gene expressions of the BMP/SMAD pathway markers SMAD1, SMAD4, SMAD5, and BMPR1A were demonstrated by RT-PCR. (b) Protein expressions of SMAD4 and BMPR1A were examined by western blot. ^*∗*^*P* < 0.05, compared to the control group.

To explore the molecular mechanisms of improved osteogenesis of BMSCs in Mg ions/RAW264.7 cells-conditioned media, the BMP/SMAD signaling pathway was studied. RT-PCR results showed that the gene expressions of SMAD4, SMAD5, and BMPR1A were increased significantly with all concentrations of Mg ions on day 3 (*P* < 0.05, Figure 7(a)). Furthermore, on day 1, the conditioned medium with 100 mg/L Mg ions caused a significant increase in the gene expression of SMAD4 and BMPR1A (*P* < 0.05, Figure 7(a)). However, gene expression of SMAD1 had no obvious change in all groups. The protein expressions of SMAD4 and BMPR1A were further confirmed by western blot. The result showed that the protein levels of SMAD4 and BMPR1A significantly enhanced in the conditioned medium with 10 and 100 mg/L Mg ions on day 3 (*P* < 0.05, Figure 7(b)). However, on day 7, 100 mg/L Mg ions/RAW264.7 cells-conditioned media also significantly upregulated the protein expression of SMAD4 and BMPR1A (*P* < 0.05, Figure 7(b)).

## 4. Discussion

In this study, the osteoimmunomodulatory properties of Mg ions with the involvement of macrophages and BMSCs were systematically investigated. Our results showed that microscale Mg ions (100 mg/L) possess the osteoimmunomodulatory property that favors bone formation. More specifically, microscale Mg ions induced the M2 phenotype changes of macrophages and release of anti-inflammatory cytokines by inhibiting the TLR-NF-*κ*B signaling pathway. Mg ions stimulated the expression of osteoinductive molecules in macrophages, and Mg ions/macrophage-conditioned medium promoted osteogenesis of BMSCs, most likely through the BMP/SMAD signaling pathway. These findings indicated that manipulating Mg ion concentration can endow the Mg scaffold with favorable osteoimmunomodulatory properties, thereby providing the fundamental evidence for the development and modification of Mg-based bone biomaterials.

Mg scaffold is a promising bone substitute due to its excellent mechanical properties and biocompatibility [3, 4, 17]. However, Mg is a highly reactive metal and corrodes quickly, thereby causing massive inflammatory reaction *in vivo* [6]. We inferred that the ionic concentration of the Mg scaffold is a key factor that determines the osteoimmunomodulatory property of biomaterials. However, a previous study showed that coating of the Mg scaffolds with *β*-TCP greatly decreased the concentration of Mg ions in solution (195.4 ± 0.86 mg/L) compared to the Mg scaffolds (1021 ± 2.13 mg/L) [9]. Mg-*β*-TCP scaffold has been proven to induce macrophages expressing the M2 surface marker, CD163, and anti-inflammatory cytokines (IL-1ra) [9]. Therefore, we hypothesized that the anti-inflammatory effects of Mg-*β*-TCP are attributed to the lower concentration of Mg ions. Our present study however demonstrated that microscale Mg ions (100 mg/L) induce a shift toward the M2 phenotype of macrophage with increased gene expression of the surface marker, CD206, and reduced the M1 phenotype marker, CCR7. Microscale Mg ions (100 mg/L) also increased the gene expression of the anti-inflammatory cytokines, IL-10 and IL-1ra, and decreased the important inflammatory cytokine, TNF-*α*. Although the inflammatory cytokines of IL-6 and IL-1*β* increased slightly, it most likely occurred with the high concentration of Mg ions (500 mg/L). Consistently, a previous study by Sugimoto et al. showed that MgSO_4_ at a concentration of 60 mg/L decreased inflammatory cytokine production of IL-6 and TNF-*α* by inhibiting the TLR receptor pathway [18], which is approximately the same concentration of Mg ion used in our study (100 mg/L). These findings indicate that the microscale Mg ions can induce macrophage polarization toward the M2 extremity and create an anti-inflammatory microenvironment for bone regeneration.

Notably, toll-like receptor (TLR) signaling is an essential pathway in the innate immune response, through which macrophages recognize foreign antibody and initiate antigen-specific adaptive immune response [19, 20]. Activation of TLR signaling is mediated by a unique interaction between TIR domain-containing cytosolic adapters which include MyD88 and TIR domain-containing adapter-inducing IFNb (TRIF) also known as toll-like receptor adapter molecule (Ticam) [20]. Importantly, upon ligand binding, TLR leads to the activation of NF-*κ*B pathways to elicit the expression of inflammatory cytokines [21]. In most cell types, NF-*κ*B is bound to its inhibitor, I-*κ*B, and resides in the cytoplasm as an inactive NF-*κ*B/I-*κ*B complex [22]. However, the activated form of NF-*κ*B is a heterodimer of the p65 subunit associated with p50 or p52 subunit, and p65/p50 or p65/p52 heterodimer migrates into the nucleus and initiates transcription of the inflammatory genes [22]. In the present study, the gene expression of Myd88, Ticam1, and Ticam2 were downregulated and the NF-*κ*B inhibitor, I*κ*B-*α*, was upregulated in the 100 and 500 mg/L Mg ion groups. Such finding would indicate that the microscale Mg ions dampen the inflammatory response potentially by inhibiting the TLR-NF-*κ*B pathway. Similarly, a previous study by Sugimoto et al. showed that MgSO_4_ decreases TLR-mediated cytokine production in monocytes by increasing I*κ*B-*α* levels and downregulating NF-*κ*B p65 levels [18]. However, in our study, protein expression of NF-*κ*B p65 showed no alteration in the Mg ion groups. Hence, we inferred that Mg ions might inhibit other components in the NF-*κ*B pathway in macrophages, such as p50 or p52. This discrepancy might be due to the diverse inflammatory cell types and different experimental conditions.

Subsequently, we sought to clarify whether the modification of macrophages by Mg ions would influence osteogenesis of BMSCs. Therefore, Mg ions/RAW264.7 cell-conditioned media were utilized for osteogenic differentiation of BMSCs.

Importantly, RUNX2 is a key transcription factor of osteoblast differentiation [23]. ALP is a well-known marker for pre-osteoblast differentiation and osteoblast mineralization [24]. In addition, OPN and OCN are important genes in the process of mineral deposition [24]. The result showed that when stimulated with conditioned media of 100 mg/L Mg ions, BMSCs resulted in a significant enhancement in ALP activity and osteogenic genes (Runx-2, ALP, OPN, and OCN), which would indicate that Mg ions promote osteogenesis of BMSCs through macrophage regulation.

Although microscale Mg ions have been shown to transit macrophages phenotype into M2, the molecular mechanisms whereby M2 macrophage influences osteogenesis are yet to be established. We hypothesized that the M2 macrophage may promote osteogenesis of BMSCs through paracrine function. Notably, a previous study reported that the M2 phenotype secretes osteoinductive and osteogenic cytokines such as BMP-2 and VEGF [14, 25]. Among the BMP family members, BMP-2 is a potent osteoinductive agent [26–28] and VEGF is an important proangiogenic factor that binds to VEGFR and initiate angiogenic cascade [29]. In the process of bone formation, angiogenesis and osteogenesis are coupled with each other as the function of VEGF and BMP-2 has been found to be closely related and synergistic [30, 31]. Indeed, our study demonstrated that Mg ions upregulated the gene expression of BMP-2 and VEGF in macrophages. We inferred that the upregulation of BMP-2 might activate the BMP-2/SMAD signaling pathway in BMSCs which is the key pathway for osteogenic differentiation. In this pathway, BMP-2 binds with BMPR2 and then recruits BMPRA1 [32]. Subsequently, phosphorylation of SMAD1/5/8 is triggered, which sequentially causes dimer complex to form with SMAD4. The complexes translocate into the cell nucleus to induce transcription of the osteogenic gene, Runx2 [32]. The result however showed the activation of BMP-2/SMAD signaling in BMSCs as demonstrated by the upregulation of SMAD4 and BMPR1A at both gene and protein levels. Therefore, it is reasonable to infer that microscale Mg ions trigger the phenotype switches of macrophages into M2 by inhibiting the TLR-NF-*κ*B signaling pathway and, as a result, causes the upregulation of anti-inflammatory cytokines (IL-10 and IL-1ra). Furthermore, the microscale Mg ions stimulate macrophages to upregulate VEGF and BMP-2 expression, which activate the BMP-2 pathway in BMSCs, thereby enhancing osteogenic differentiation of stem cells. The present study proposed that manipulating Mg concentration in bone biomaterial could regulate the immune environment that positively influences osteogenesis and avoids the destructive inflammatory reaction caused by the Mg-based biomaterial.

Apart from secretion of VEGF and BMP-2, excessive M2 macrophages have been reported to secrete fibrous agents, such as TGF-*β*s, resulting in pathological fibrosis, formation of scar tissue, or delayed wound healing [14, 25, 33]. TGF-*β*1 is a potent cytokine to promote fibroblast proliferation [34], and TGF-*β*3 induces the synthesis of extracellular matrix (ECM) protein, such as type I collagen, fibronectin, proteoglycans, and laminin [35]. In this study, we found the downregulation of TGF-*β*3 in Mg ion groups, which indicated that maybe stimulating macrophages with microscale Mg ions could not induce pathological fibrosis.

## 5. Conclusions

In summary, controlling the releasing concentration of Mg ions (approximately 100 mg/L) conquers the detrimental osteoimmunomodulatory properties of Mg-based biomaterials, causing them to be more favorable towards osteogenesis of BMSCs. Specifically, microscale Mg ions induced M2 macrophage phenotype switches and produced an anti-inflammatory environment most likely through the inhibition of the TLR-NF-*κ*B signaling pathway. Microscale Mg ions stimulate macrophage expression of BMP-2 and activate the BMP-2 signaling pathway in BMSCs, thereby enhancing osteogenic differentiation. Therefore, manipulating the concentration of Mg ions in Mg-based bone scaffolds endows biomaterials with favorable osteoimmunomodulatory properties. The present study provides fundamental evidence and proposes novel strategies for the development or modification of advanced Mg-based bone biomaterials using stem cells.

## Figures and Tables

**Table 1:** Primer pairs used in the qRT-PCR.

Gene	Primer sequences
CD206	Forward: 5′-AGACGAAATCCCTGCTACTG-3′
	Reverse: 5′-CACCCATTCGAAGGCATTC-3′
CCR7	Forward: 5′-ATGACGTCACCTACAGCCTG-3′
	Reverse: 5′-CAGCCCAAGTCCTTGAAGAG-3′
IL-1ra	Forward: 5′-CTCCAGCTGGAGGAAGTTAAC-3′
	Reverse: 5′-CTGACTCAAAGCTGGTGGTG-3′
IL-10	Forward: 5′-GAGAAGCATGGCCCAGAAATC-3′
	Reverse: 5′-GAGAAATCGATGACAGCGCC-3′
IL-1*β*	Forward: 5′-TGGAGAGTGTGGATCCCAAG-3′
	Reverse: 5′-GGTGCTGATGTACCAGTTGG-3′
IL-6	Forward: 5′-ATAGTCCTTCCTACCCCAATTTCC-3′
	Reverse: 5′-GATGAATTGGATGGTCTTGGTCC-3′
IL-18	Forward: 5′-TGGCCGACTTCACTGTACAAC-3′
	Reverse: 5′-TGGGGTTCACTGGCACTTTG-3′
TNF-*α*	Forward: 5′-CTGAACTTCGGGGTGATCGG-3′
	Reverse: 5′-GGCTTGTCACTCGAATTTTGAGA-3′
Myd88	Forward: 5′-AGGTAAGCAGCAGAACCAGG -3′
	Reverse: 5′-TGTCCTAGGGGGTCATCAAGG-3′
Ticam1	Forward: 5′-AGATGGTTCAGCTGGGTGTC-3′
	Reverse: 5′-TGGAGTCTCAAGAAGGGGTTC-3′
Ticam2	Forward: 5′-CTTGGCGCTGCAAACCATC-3′
	Reverse: 5′-GCCTCTCAAATACAGACTCCCG-3′
TGF-*β*1	Forward: 5′-GTGGAAATCAACGGGATCAGC-3′
	Reverse: 5′-CAGCAGTTCTTCTCTGTGGAGC-3′
TGF-*β*3	Forward: 5′-CAACACCCTGAACCCAGAG-3′
	Reverse: 5′-CTT CACCACCATGTTGGACAG-3′
BMP-2	Forward: 5′-GCTCCACAAACGAGAAAAGC-3′
	Reverse: 5′-AGCAAGGGGAAAAGGACACT-3′
BMP-6	Forward: 5′-TGGCAGGACTGGATCATTGC-3′
	Reverse: 5′-ACCAAGGTCTGTACAATGGCG-3′
VEGF	Forward: 5′-GTCCCATGAAGTGATCAAGTTC-3′
	Reverse: 5′-TCTGCATGGTGATGTTGCTCTCTG-3′
GAPDH (mouse)	Forward: 5′-TGACCACAGTCCATGCCATC-3′
	Reverse: 5′-GACGGACACATTGGGGGTAG-3′
Runx-2	Forward: 5′-TCTTTTGGGATCCGAGCACC-3′
	Reverse: 5′-ATCTCCACCATGGTGCGGTT-3′
ALP	Forward: 5′-CCA TTT CAG CCT CAG GAT CG-3′
	Reverse: 5′-TGG CCA CGT TGG TGT TGA GT-3′
OPN	Forward: 5′-CCAAGCGTGGAAACACACAGCC-3′
	Reverse: 5′-GGCTTTGGAACTCGCCTGACTG-3′
OCN	Forward: 5′-GCCCTGACTGCATTCTGCCTCT-3′
	Reverse: 5′-TCACCACCTTACTGCCCTCCTG-3′
SMAD4	Forward: 5′- TACCACCATAACAGCACTAC-3′
	Reverse: 5′-GAACACCAATATTCAGGAGC-3′
SMAD5	Forward: 5′-GTACTATGAACTGAACAACGG-3′
	Reverse: 5′-TATAGATGGACACCTTTCCC-3′
SMAD1	Forward: 5′-GAGATCAATAGAGGAGATGTTC -3′
	Reverse: 5′-TCGGTTCTTATTGTTGGAAG-3′
BMPR1A	Forward: 5′-GACACGTGCGAATTGGACAATG-3′
	Reverse: 5′-CGTCTGATTTCATACCAGTAC-3′
GAPDH (rat)	Forward: 5′-TCAGCAATGCCTCCTGCAC-3′
	Reverse: 5′-TCTGGGTGGCAGTGATGGC-3′

## Data Availability

The data used to support the findings of this study are available from the corresponding author upon request.
